# Supplementary material for: Causal interplay between lactose intolerance and gut microbiota: a combined bidirectional Mendelian randomization and in vivo validation study
Source: Front Nutr. 2026 Jun 1;13:1803337. doi: 10.3389/fnut.2026.1803337 (PMC13265576; doi:10.3389/fnut.2026.1803337)
Supplement: Supplementary file 1 [file Data_Sheet_1.zip › supplementary materials/Forward/forest plot/ebi-a-GCST90027780.finngen_R12_E4_LACTONAS.pdf]

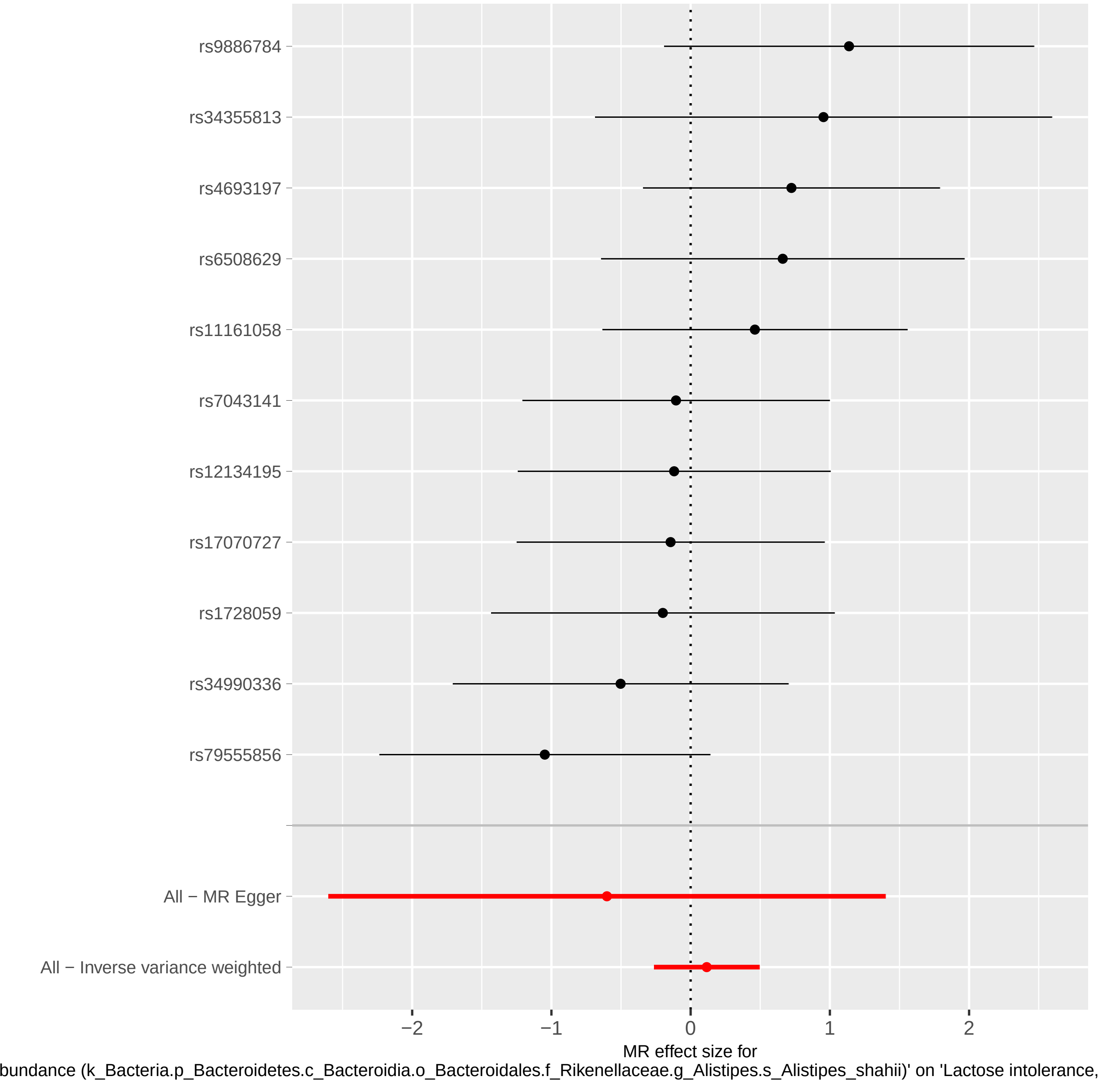
rs9886784
rs34355813
rs4693197
rs6508629
rs11161058
rs7043141
rs12134195
rs17070727
rs1728059
rs34990336
rs79555856
All − MR Egger
All − Inverse variance weighted
−2
−1
0
1
2
MR effect size for
bundance (k_Bacteria.p_Bacteroidetes.c_Bacteroidia.o_Bacteroidales.f_Rikenellaceae.g_Alistipes.s_Alistipes_shahii)' on 'Lactose intolerance,
